# Anti-oncogenic effects of SOX2 silencing on hepatocellular carcinoma achieved by upregulating miR-222-5p-dependent CYLD via the long noncoding RNA CCAT1

**DOI:** 10.18632/aging.103797

**Published:** 2021-03-22

**Authors:** Jian Pu, Xianjian Wu, Yi Wu, Zesheng Shao, Chunying Luo, Qianli Tang, Jianchu Wang, Huamei Wei, Yuan Lu

**Affiliations:** 1 Department of Hepatobiliary Surgery, Affiliated Hospital of Youjiang Medical University for Nationalities, Baise 533000, P.R. China; 2 Graduate College of Youjiang Medical University for Nationalities, Baise 533000, P.R. China; 3 Department of Pathology, Affiliated Hospital of Youjiang Medical University for Nationalities, Baise 533000, P.R. China

**Keywords:** SOX2, CCAT1, EGFR, miR-222-5p, CYLD

## Abstract

In this study, we determined the involvement of SOX2 and its downstream signaling molecules in hepatocellular carcinoma (HCC) progression. We carried out lentiviral transfection in HepG2 cells to determine the roles of SOX2, CCAT1, EGFR, miR-222-5p, and CYLD in HepG2 cells. We first determined the interaction between SOX2 and CCAT1 and that between miR-222-5p and CYLD and their effect on tumor growth *in vivo* was analyzed in HCC-xenograft bearing nude mice xenografts. SOX2 and CCAT1 were highly expressed in HCC tissues and HepG2 cells. SOX2 bound to the regulatory site of CCAT1. Silencing of SOX2 or CCAT1 inhibited HepG2 cell proliferation, migration, and invasion as well as decreased the expression of CCAT1 and EGFR. CCAT1 silencing reduced EGFR expression, but EGFR expression was increased in HCC tissues and HepG2 cells, which promoted proliferation, migration, and invasion *in vitro*. EGFR upregulated miR-222-5p, leading to downregulation of CYLD. miR-222-5p inhibition or CYLD overexpression repressed cell functions in HepG2 cells. SOX2 silencing decreased CCAT1, EGFR, and miR-222-5p expression but increased CYLD expression. Loss of SOX2 also reduced the growth rate of tumor xenografts. In summary, SOX2-mediated HCC progression through an axis involving CCAT1, EGFR, and miR-222-5p upregulation and CYLD downregulation.

## INTRODUCTION

Hepatocellular carcinoma (HCC) is currently the fourth most common cause of cancer death worldwide [1, 2] and accounts for over 80% of all primary liver cancer [3]. Risk factors for HCC include fatty liver disease, heavy alcohol consumption, hepatitis B and C infection, and exposure to dietary toxins [4, 5]. There are wide regional differences in the incidence of HCC, with the majority of cases occurring in countries at a low to medium stage of economic development [6]. In contrast to the decreasing incidences in many types of cancers, the incidence of HCC remains on an upward trend, with the case number worldwide having increased by 4.6% from 2005 to 2015 [7]. In spite of considerable advancement in its prevention and early detection [8], this increasing incidence of HCC must be attributable to some still unknown aspects of its pathogenesis. This state of affairs calls for investigation aiming to provide a better understanding of mechanisms underlying HCC.

Sex determining region Y-box 2 (SOX2) was first characterized as a transcription factor involved in stem cell proliferation and maintenance [9]. Recently, SOX2 has been linked to cancer progression due to its effects on cell proliferation, invasion, and migration properties [10], with well-established involvement in prostate and cervical cancers [11, 12], and likewise in the case of HCC development [13–15]. Previous work has shown that activation of the PI3K/AKT signaling pathway could up-regulate SOX2 expression of human HCC stem cells [14]. Furthermore, high SOX2 expression is associated with the prediction of poor survival in HCC patients [16]. These observations call for examination of the downstream mechanism of SOX2 in HCC.

Long non-coding RNA colon cancer-associated transcript 1 (lncRNA CCAT1) is documented to promote a number of cancers, including colorectal, gastric, gallbladder, and HCC [17–20]. CCAT1 functions as an oncogene during HCC development by promoting cell proliferation and migration [21]. Of note, CCAT1 has been linked to SOX2 as a coactivator in squamous cell carcinoma, and CCAT1 binds to epidermal growth factor receptor (EGFR) super-enhancer to stimulate EGFR expression by forming a complex with TP65 and SOX2 [22, 23]. EGFR is a transmembrane receptor for ligands of the epidermal growth factor family extracellular proteins [24], and activation of EGFR is well-known to enhance the growth of HCC and various other cancers by increasing their invasiveness [25–27].

microRNAs (miRs) have recently emerged as factors involved in EGFR-mediated functions in cancer cells [28, 29]. miR-222-5p is implicated in various tumor types, including thyroid and pancreatic cancer [30, 31]. A recent study reported high miR-222-5p expression in HCC tissues and cells [32]. Importantly, our present interrogation of the TargetScan website predicted the binding site between miR-222-5p and the deubiquitinating enzyme CYLD that is implicated in the familial skin tumor cylindromatosis. In quite a number of cancers, downregulation of CYLD results in impaired cell proliferation and survival, which indicatives an antitumor potential of CYLD [33]. Additionally, a study conducted by Ni et al. demonstrated that CYLD was targeted by miR-362-5p to induce hepatocarcinogenesis [34]. Therefore, we formulated the hypothesis that SOX2-mediated CCAT1 might correlate to HCC development via the EGFR/miR-222-5P/CYLD axis. Therefore, we undertook a series of studies *in vitro* and in HCC-bearing nude mice to explore the specific mechanisms of CCAT1 in HCC with the involvement of SOX2, EGFR, miR-222-5P and CYLD.

## RESULTS

### SOX2-mediated CCAT1 promotes EGFR expression in HepG2 cells

**Figure 1:**
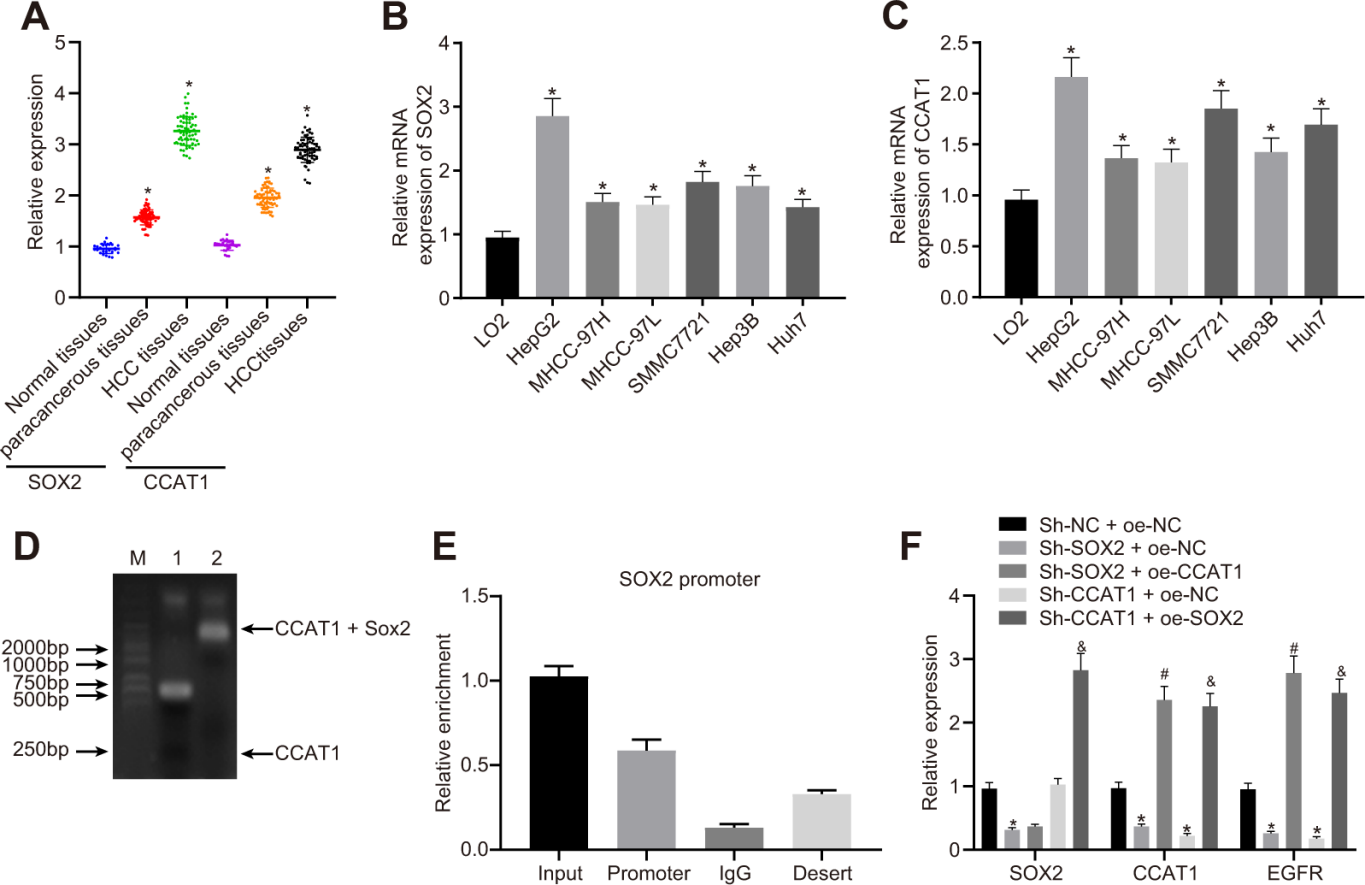
**SOX2-mediated lncRNA CCAT1 promotes EGFR expression in HepG2 cells.** (**A**) SOX2 and CCAT1 expression in normal liver (n = 28), para-cancerous (n = 68) and HCC tissues (n = 68) detected by RT-qPCR normalized to β-actin. (**B**) SOX2 mRNA expression in human HCC cell lines detected by RT-qPCR. (**C**) CCAT1 expression in human HCC cell lines detected by RT-qPCR. (**D**) Interaction of SOX2 with CCAT1 in HepG2 cell line detected using EMSA. (**E**) ChIP-qPCR results of the binding of SOX2 to CCAT1 promoter region in HepG2 cell line. (**F**) SOX2 expression and CCAT1 and EGFR mRNA expression after alteration of SOX2 and CCAT1 in HepG2 cells detected by RT-qPCR. * *p* < 0.05 *vs.* normal liver cell, LO2 or HepG2 treated with sh-NC + oe-NC. # *p* < 0.05 *vs.* HepG2 treated with sh-SOX2 + oe-NC. & *p* < 0.05 *vs.* HepG2 treated with sh-SOX2 + oe-NC. Data are expressed as mean ± standard deviation. Data comparisons among multiple groups were performed by one-way ANOVA and Tukey’s *post hoc* test.

To explore the relationship among SOX2, CCAT1, and EGFR in HCC cells, we measured their expression in HCC tissues and cells. The expression of SOX2 and CCAT1 in HCC tissues was significantly higher than that in para-cancerous tissues and normal liver tissues (Figure 1A). Similarly, all six human HCC cell lines (HepG2, MHCC-97H, MHCC-97L, SMMC7721, Hep3B, and Huh7) had higher SOX2 (Figure 1B) and CCAT1 (Figure 1C) expression than the normal human liver cell line LO2. Because SOX2 and CCAT1 expression was the highest in HepG2 cells, these were selected for further experiments.

Existing literature has reported that SOX2-mediated activation of CCAT1 super-enhancer could promote the expression of CCAT1 [22]. Our results of EMSA displayed that SOX2 could bind to the CCAT1 promoter. Furthermore, we also showed by ChIP-qPCR analysis that SOX2 could bind to the promoter region of CCAT1, consistently suggesting that SOX2 binds to the CCAT1 promoter region to elevate the expression of CCAT1 (Figure 1D, 1E). Previous work reports that CCAT1, TP63, and SOX2 form a complex that binds to the EGFR enhancer region to promote EGFR expression [35]. Therefore, to test this we silenced or overexpressed SOX2 and CCAT1 in HepG2 cells, and then measured SOX2, CCAT1, and EGFR expression. The SOX2 silencing significantly reduced CCAT1 expression, whereas after knockdown of CCAT1, SOX2 expression unaffected in HepG2 cells. Furthermore, knockdown of SOX2 along with overexpression of CCAT1 did not affect SOX2 expression compared with only knockdown of SOX2. However, CCAT1 knockdown with SOX2 overexpression significantly increased CCAT1 expression compared to the CCAT1 knockdown. These results affirmed that CCAT1 is a downstream target of SOX2 in HCC. Moreover, knockdown of SOX2 or CCAT1 reduced EGFR mRNA levels, whereas SOX2 knockdown with CCAT1 overexpression increased EGFR mRNA levels. CCAT1 knockdown and SOX2 overexpression produced similar results (Figure 1F). These results suggest that SOX2 mediates effects of CCAT1 to promote EGFR expression in HCC cells.

### SOX2 and CCAT1 promote HCC cell proliferation, migration, and invasion through up-regulation of EGFR

**Figure 2:**
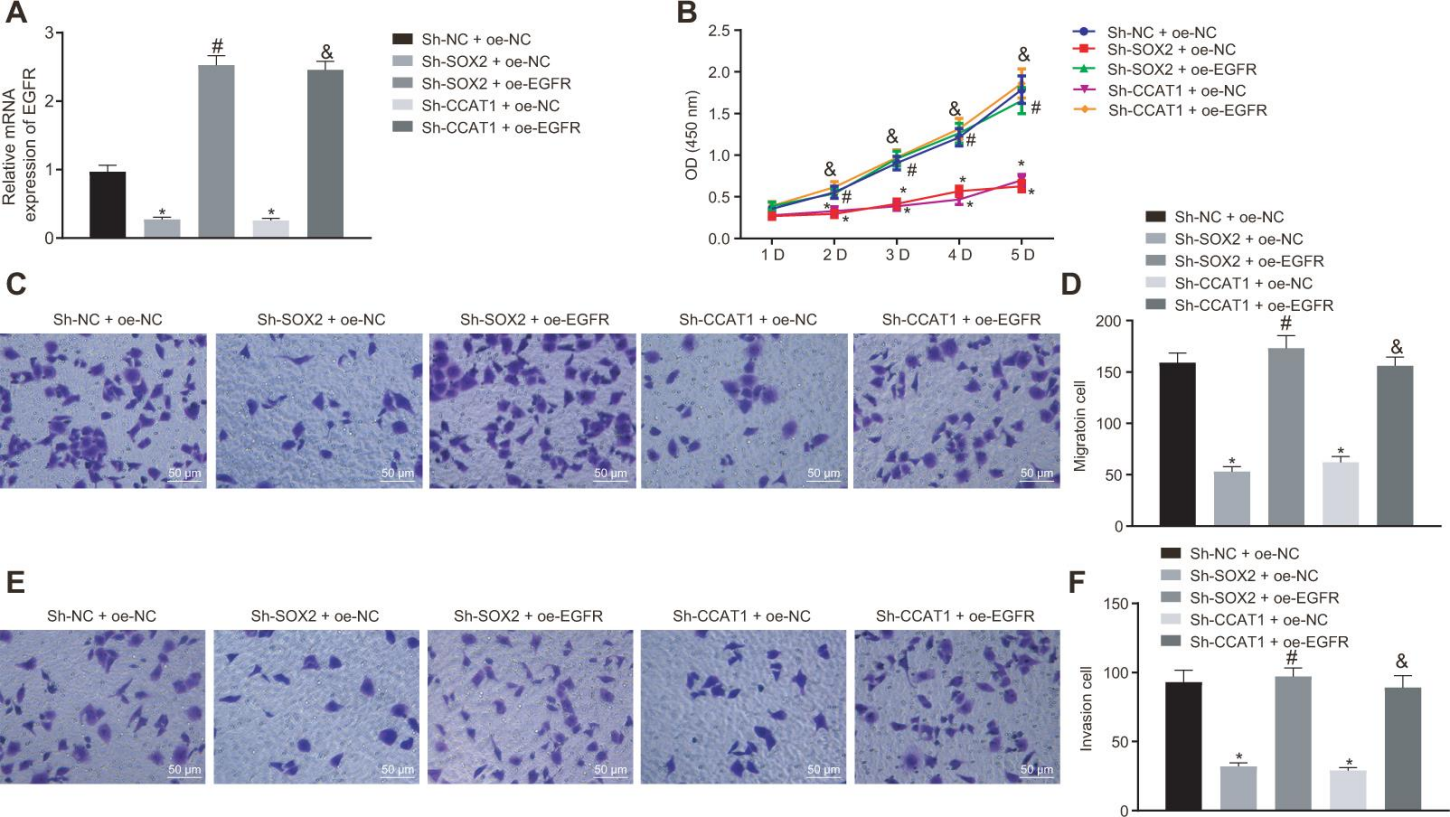
**Silencing of SOX2 and CCAT1 decreases EGFR expression to suppress HepG2 cell proliferation, migration, and invasion.** HepG2 cells were treated with sh-NC + oe-NC, sh-SOX2 + oe-NC, sh-CCAT1 + oe-NC, sh-SOX2 + oe-EGFR or sh-CCAT1 + oe-EGFR. (**A**) mRNA expression of EGFR in HepG2 cells detected by RT-qPCR normalized to β-actin. (**B**) Cell viability determined by MTT assay. (**C**, **D**) cell migration determined by Transwell assay (200 ×). (**E**, **F**) Cell invasion determined by Transwell assay (200 ×). * *p* < 0.05 *vs.* HepG2 treated with sh-NC + oe-NC. # *p* < 0.05 *vs.* HepG2 treated with sh-SOX2 + oe-NC. & *p* < 0.05 *vs.* HepG2 treated with sh-SOX2 + oe-NC. Data were expressed as mean ± standard deviation. Data from multiple groups were compared by one-way ANOVA and Tukey’s *post hoc* test. Data were compared between groups at different time points by repeated measures ANOVA and Bonferroni-corrected *post hoc* testing.

To explore how SOX2 and CCAT1 regulated HCC cell functions via EGFR, we silenced SOX2 and CCAT1 and overexpressed EGFR in HepG2 cells and then measured cell proliferation, invasion, and migration. SOX2 or CCAT1 knockdown reduced expression of EGFR, which was rescued by oe-EGFR treatment (Figure 2A). As displayed in Figure 2B–2F, SOX2 or CCAT1 knockdown contributed to declining HepG2 cell viability, migration, and invasion, which were rescued by additional EGFR overexpression. The above-mentioned results suggest that SOX2/CCAT1 promotes proliferation, migration, and invasion of HCC cells by activating EGFR.

### EGFR silencing suppresses cell malignant phenotype by downregulating miR-222-5p in HepG2 cells

**Figure 3:**
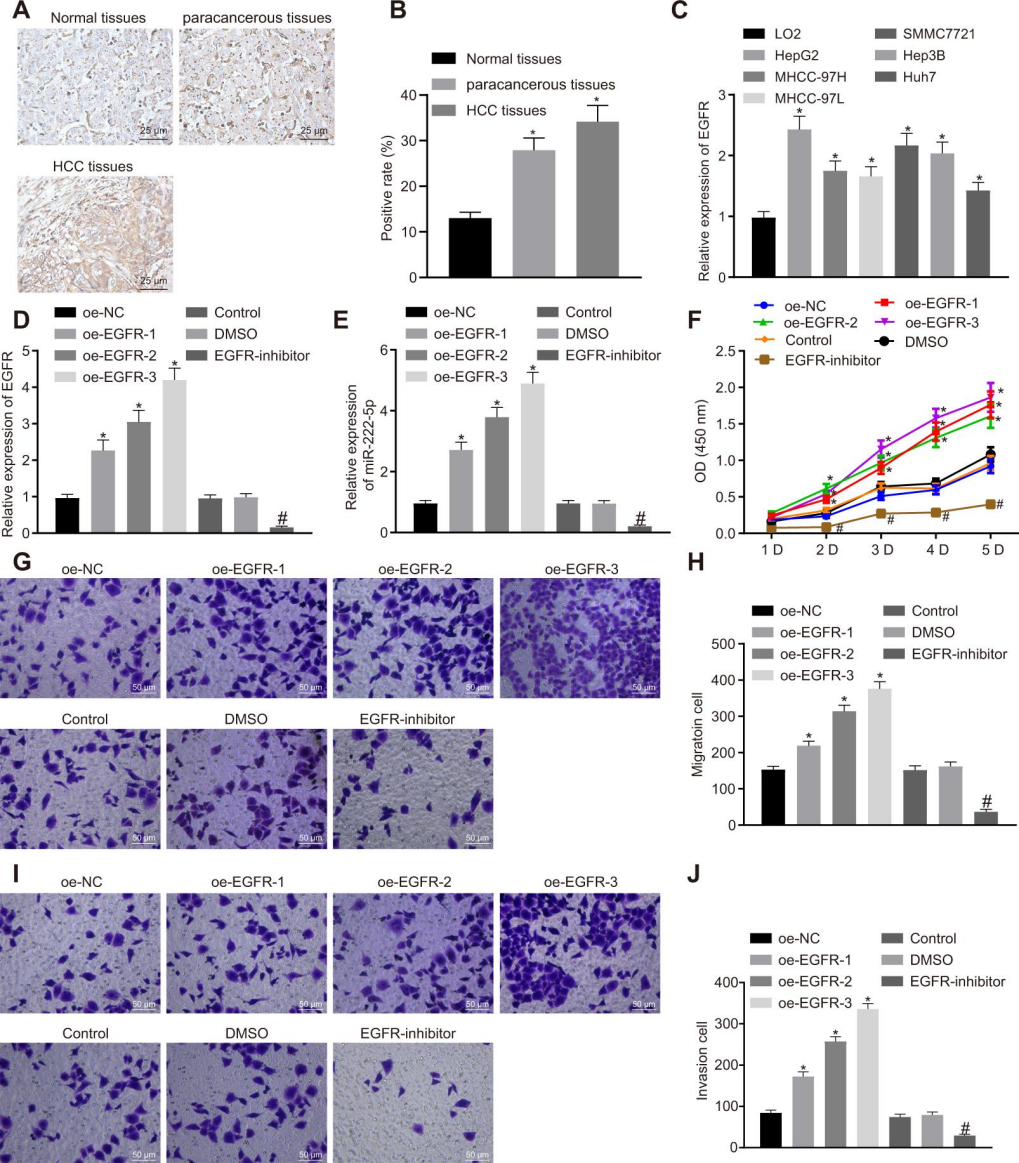
**EGFR induces cell proliferation, migration and invasion via miR-222-5p upregulation in HepG2 cells.** (**A**) Representative immunohistochemistry micrographs showing EGFR expression in normal liver (n = 28), para-cancerous (n = 68) and HCC tissues (n = 68) (400 ×). (**B**) EGFR positive expression in normal liver (n = 28), para-cancerous (n = 68) and HCC tissues (n = 68) determined by immunohistochemistry. (**C**) EGFR mRNA expressions in human HCC cell lines detected by RT-qPCR normalized to β-actin. HepG2 cells were treated with oe-NC, oe-EGFR-1, oe-EGFR-2, oe-EGFR-3, DMSO or EGFR-inhibitor. (**D**) EGFR expression in HepG2 cells detected by RT-qPCR. (**E**) miR-222-5p expression in HepG2 cells detected by RT-qPCR normalized to U6. (**F**) Cell viability determined by MTT assay. (**G**, **H**) Cell migration determined by Transwell assay (200 ×). (**I**, **J**) Cell invasion determined by Transwell assay (200 ×). * *p* < 0.05 *vs.* normal liver cell, LO2 or HepG2 cells treated with oe-NC; # *p* < 0.05 *vs.* HepG2 treated with DMSO. Data are expressed as mean ± standard deviation. Data from multiple groups were compared by one-way ANOVA and Tukey’s *post hoc* test. Data were compared between groups at different time points by repeated measures ANOVA and Bonferroni *post hoc* test.

Previous work has suggested that EGFR could elevate the expression of miR-222-5p [29]. Therefore, we set about to explore the downstream mechanism of EGFR in HCC. First, our immunohistochemistry study showed that the rate of EGFR positive expression was 34.2 % in HCC tissues, 27.9 % in para-cancerous tissues, and only 13.0% in normal liver tissues, indicating higher EGFR expression in HCC tissues than in para-cancerous and normal liver tissues (Figure 3A, 3B). Similarly, RT-qPCR also showed higher EGFR mRNA expression in all six human HCC cell lines than in the LO2 normal liver cell line (Figure 3C). Among them, HepG2 cells had the highest EGFR expression and were therefore selected for further experiments.

HepG2 cells cultured in a volume of X mL were treated with oe-EGFR-1 (0.5 μg), oe-EGFR-2 (1 μg), oe-EGFR-3 (1.5 μg), or EGFR inhibitor (Gefitinib, 1 μM). In transfected HepG2 cells, transfection for EGFR overexpression at the three different levels significantly increased EGFR expression in a dose-dependent manner. In contrast, EGFR inhibitor significantly reduced EGFR expression (Figure 3D). Moreover, EGFR overexpression significantly increased miR-222-5p expression in a dose-dependent manner, while EGFR inhibitor reduced its expression (Figure 3E). In the MTT assay, EGFR overexpression promoted cell viability in a dose-dependent manner while EGFR inhibitor treatment significantly reduced cell viability (Figure 3F). These effects peaked on day five in culture. In the Transwell assay, EGFR overexpression significantly elevated cell migration (Figure 3G, 3H) and invasion (Figure 3I, 3J) in a dose-dependent manner that was blocked by EGFR inhibitor. The aforementioned results demonstrate that silencing of EGFR resulted in suppression of HCC cell proliferation, migration, and invasion via miR-222-5p.

### miR-222-5p binds to and downregulates CYLD in HepG2 cells

**Figure 4:**
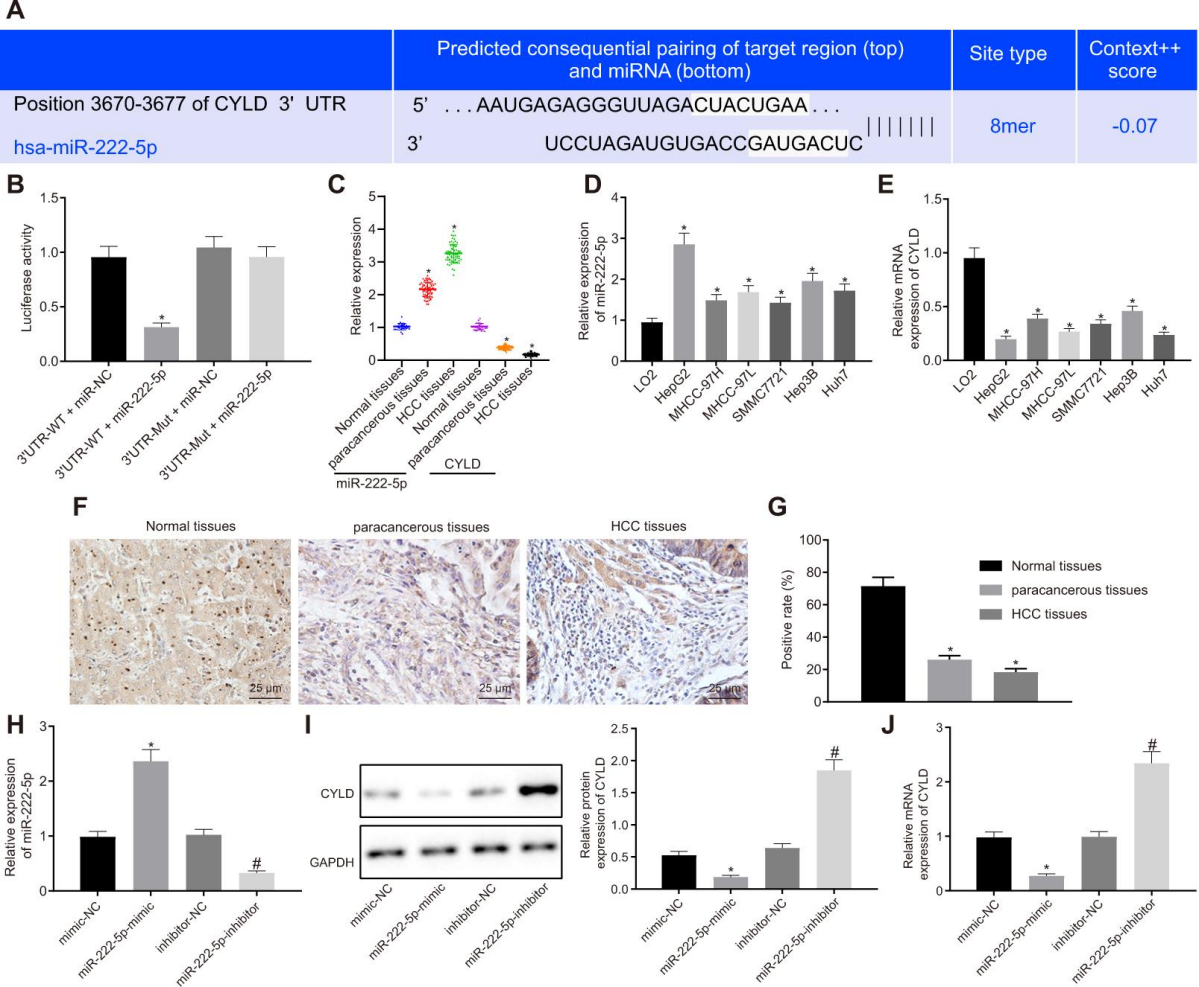
**miR-222-5p binds to and downregulates CYLD in HepG2 cells.** (**A**) Binding relationship determined by interrogation of online bioinformatics website. (**B**) Binding relationship determined by dual luciferase reporter gene assay. (**C**) miR-222-5p expression and CYLD mRNA expression in normal liver (n = 28), para-cancerous (n = 68) and HCC tissues (n = 68) detected by RT-qPCR normalized to U6 and β-actin. (**D**) miR-222-5p mRNA expression in HCC cell lines detected by RT-qPCR normalized to U6. (**E**) CYLD mRNA expression in HCC cell lines detected by RT-qPCR normalized to β-actin. (**F**) Representative micrographs showing CYLD expression in normal liver (n = 28), para-cancerous (n = 68) and HCC tissues (n = 68) determined by immunohistochemistry (400 ×). (**G**) CYLD positive staining in normal liver (n = 28), para-cancerous (n = 68) and HCC tissues (n = 68). (**H**) miR-222-5p expression in HepG2 cells after alteration of miR-222-5p detected by RT-qPCR normalized to U6. (**I**) CYLD protein expression in HepG2 cells after alteration of miR-222-5p determined by western blot analysis normalized to GAPDH. (**J**) CYLD mRNA expression in HepG2 cells after alteration of miR-222-5p determined by RT-qPCR normalized to β-actin. * *p* < 0.05 *vs.* normal liver cell, LO2 or HepG2 treated with mimic-NC; # *p* < 0.05 *vs.* HepG2 treated with inhibitor-NC. Data are expressed as mean ± standard deviation. Data between two groups were compared by independent sample t-test, and data from multiple groups were compared by one-way ANOVA and Tukey’s *post hoc* testing.

Aiming to evaluate the downstream target gene of miR-222-5p, we interrogated the TargetScan database, which revealed a specific binding region between CYLD gene sequence and the miR-222-5p sequence (Figure 4A). The dual luciferase reporter assay exhibited a conspicuous decrease of luciferase activity in CYLD 3'UTR-WT by treatment with miR-222-5p mimic, but no such difference in luciferase activity in CYLD 3'UTR-MUT (Figure 4B), this indicating that miR-222-5p specifically binds to CYLD.

Assessment of miR-222-5p and CYLD expression in tissues and cells depicted that miR-222-5p expression in HCC tissues was significantly higher than that in normal liver tissues, while CYLD expression was lower in HCC tissues (Figure 4C). Similarly, miR-222-5p expression was significantly increased in the six HCC cell lines versus the normal liver cell line, LO2 (Figure 4D). HepG2 cells had the highest miR-222-5p expressions among all HCC cell lines. In contrast, CYLD expression was low in all six HCC cell lines, with the lowest expression in HepG2 cells (Figure 4E).

Besides, immunohistochemistry showed that the CYLD positive expression rate was 18.4 % in HCC tissues, 26.2 % in para-cancerous tissues, and 71.6 % in normal liver tissues (Figure 4F, 4G), which indicates that CYLD gave a strong immunoreactivity signal in normal tissues, but had notably lower expression in HCC and para-cancerous tissues. Moreover, we transfected HepG2 cells with miR-222-5p-mimic and miR-222-5p-inhibitor. RT-qPCR and western blot analysis (Figure 4H–4J) revealed that miR-222-5p mimic significantly decreased protein and mRNA expressions of CYLD, while miR-222-5p inhibitor increased their expression. Collectively, we find that miR-222-5p negatively targeted CYLD.

### EGFR silencing represses cell proliferation, migration, and invasion by upregulating CYLD through miR-222-5p in HepG2 cells

**Figure 5:**
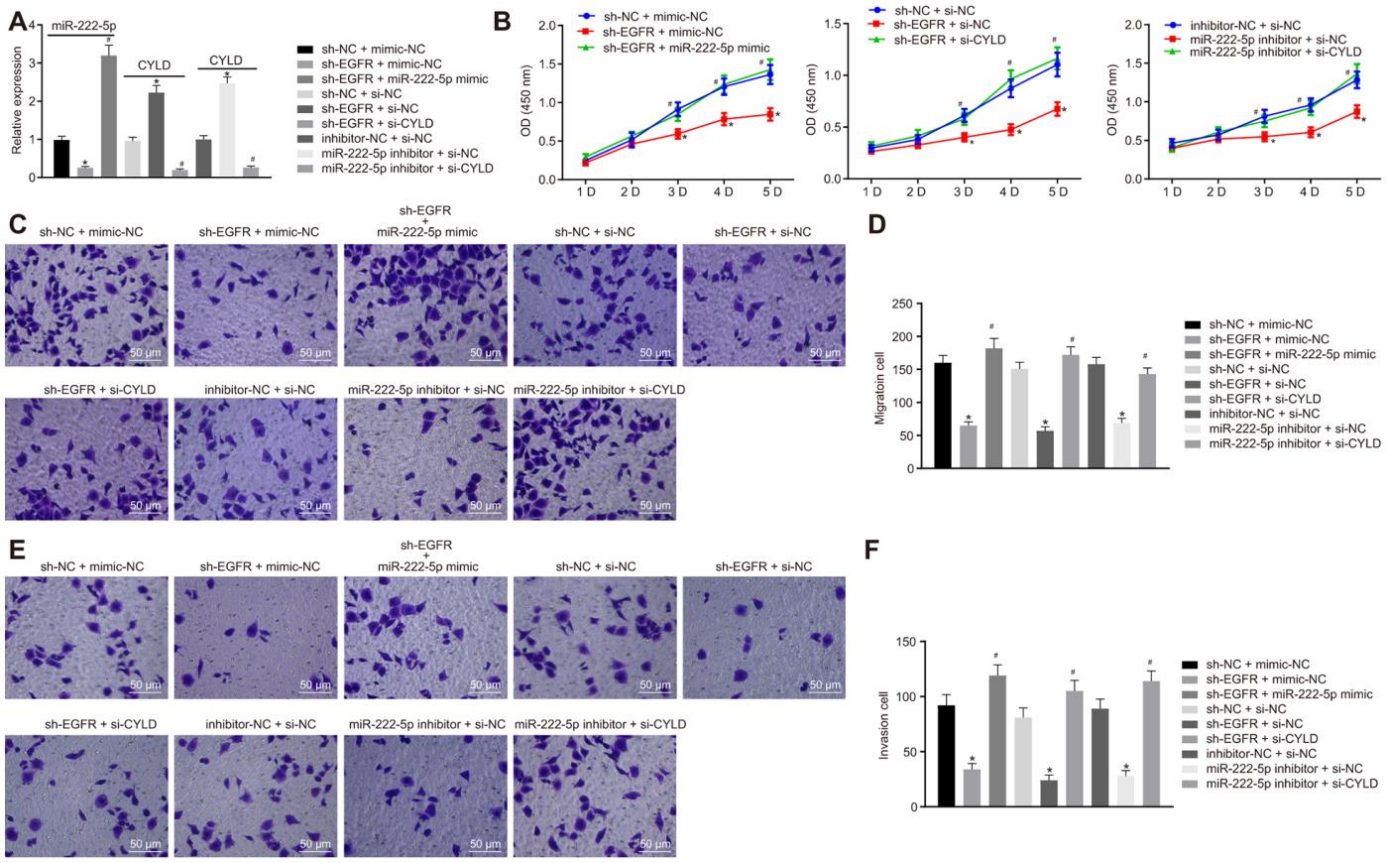
**EGFR downregulation inhibits cell proliferation, migration, and invasion by downregulating miR-222-5p-targeted CYLD in HepG2 cells.** HepG2 cells were treated with sh-NC + mimic-NC, sh-EGFR + mimic-NC, sh-EGFR + miR-222-5p mimic, sh-NC + si-NC, sh-EGFR + si-NC, sh-EGFR + si-CYLD, inhibitor-NC + si-NC, miR-222-5p inhibitor + si-NC, or miR-222-5p inhibitor + si-CYLD. (**A**) mRNA expression of miR-222-5p and CYLD in HepG2 cells detected by RT-qPCR normalized to U6 and β-actin. (**B**) Cell viability determined by MTT assay. (**C**, **D**) Cell migration determined by Transwell assay (200 ×). (**E**, **F**) Cell invasion determined by Transwell assay (200 ×). * *p* < 0.05 *vs.* HepG2 treated with sh-NC + mimic-NC, inhibitor-NC + si-NC, or sh-NC + si-NC. # *p* < 0.05 *vs.* HepG2 treated with sh-EGFR + mimic-NC, sh-EGFR + si-NC, or miR-222-5p inhibitor + si-NC. Data are expressed as mean ± standard deviation. Data from multiple groups were compared by one-way ANOVA and Tukey’s *post hoc* test. Data were compared between groups at different time points by repeated measures ANOVA and Bonferroni *post hoc* test.

We next investigated whether EGFR regulated the phenotype of HCC cells via miR-222-5p-targeting CYLD in experiments where HepG2 cells were with sh-EGFR and miR-222-5p inhibitor. RT-qPCR results showed that EGFR silencing decreased miR-222-5p expression, but enhanced CYLD expression, whereas miR-222-5p inhibitor increased CYLD expression (Figure 5A). These results show that EGFR upregulated miR-222-5p, while miR-222-5p downregulated CYLD. In other words, EGFR inhibited CYLD by mediating effects on miR-222-5p. In the MTT assay, EGFR silencing led to inhibited cell viability that was normalized by treatment with miR-222-5p mimic (Figure 5B1) or si-CYLD (Figure 5B2). Additionally, cell viability was reduced by miR-222-5p inhibitor treatment, but this effect was neutralized by silencing CYLD (Figure 5B3). In the Transwell assay, EGFR silencing decreased cell migration (Figure 5C, 5D) and invasion (Figure 5E, 5F), which were all rescued by treatment with miR-222-5p mimic or si-CYLD. The miR-222-5p inhibitor also decreased cell migration and invasion in the Transwell assay; these effects were abolished after silencing CYLD (Figure 5C–5F). In summary, EGFR silencing inhibited cell proliferation, migration, and invasion by activating CYLD via miR-222-5p in HepG2 cells.

### SOX2 silencing inhibits HepG2 cell proliferation, migration, and invasion through activating miR-222-5p

**Figure 6:**
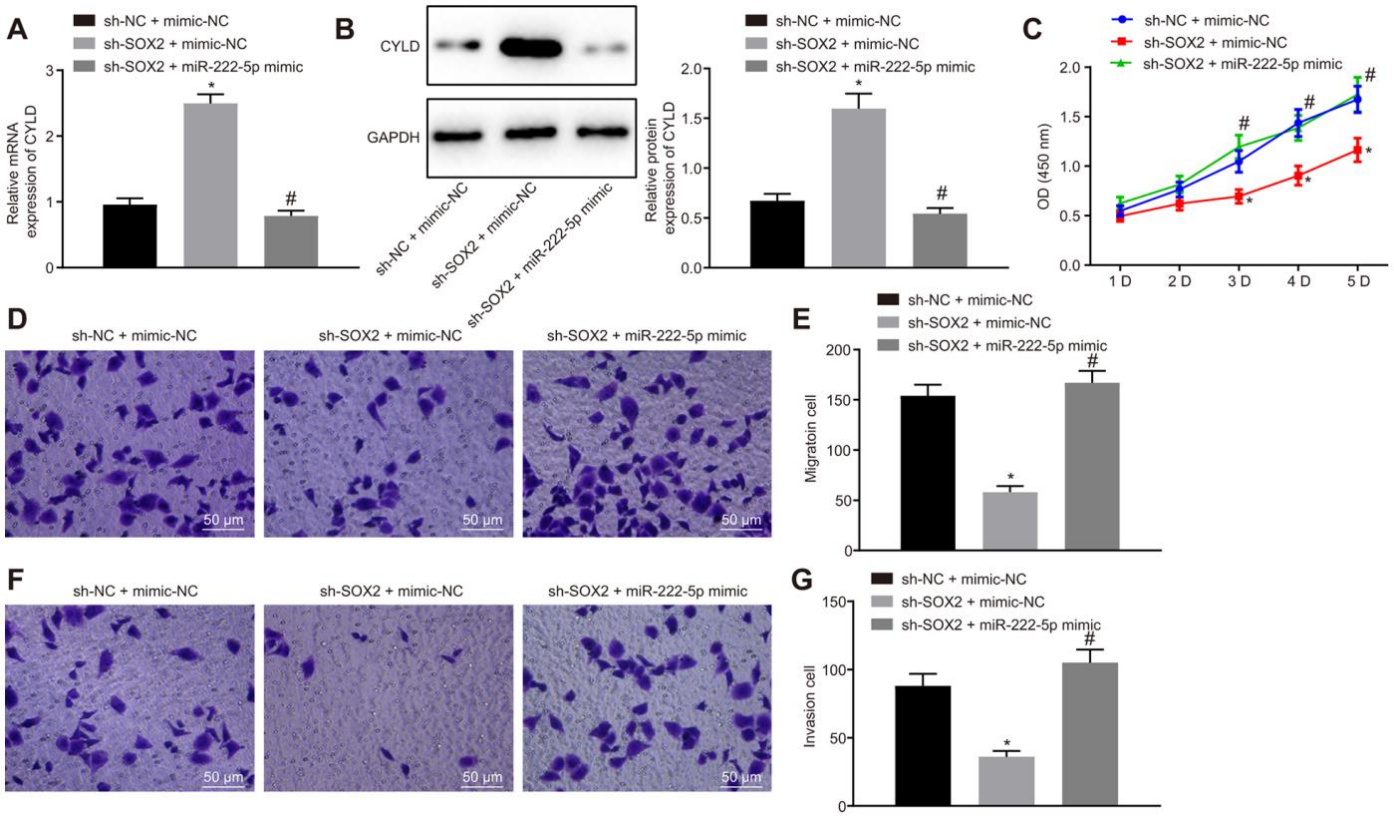
**SOX2 downregulation declines cell proliferation, migration, and invasion via miR-222-5p/CYLD in HepG2 cells.** HepG2 cells were treated with sh-NC + mimic-NC, sh-SOX2 + mimic-NC, or sh-SOX2 + miR-222-5p mimic. (**A**) CYLD mRNA expression in HepG2 cells detected by RT-qPCR normalized to β-actin. (**B**). CYLD protein expression in HepG2 cells determined by western blot analysis normalized to GAPDH. (**C**) Cell viability determined by MTT assay. (**D**, **E**) Cell migration determined by Transwell assay (200 ×). (**F**, **G**) Cell invasion determined by Transwell assay (200 ×). * *p* < 0.05 *vs.* HepG2 treated with sh-NC + mimic-NC. # *p* < 0.05 *vs.* HepG2 treated with sh-SOX2 + mimic-NC. Data are expressed as mean ± standard deviation. Data from multiple groups were compared by one-way ANOVA and Tukey’s *post hoc* test. Data were compared between groups at different time points by repeated measures ANOVA and Bonferroni *post hoc* test.

Moreover, we silenced SOX2 and overexpressed miR-222-5p in HepG2 cells to identify whether SOX2 regulated miR-222-5p. SOX2 silencing resulted in elevation of both of CYLD mRNA (Figure 6A) and protein (Figure 6B) expression, both of which were normalized by miR-222-5p mimic. In the MTT (Figure 6C) and Transwell (Figure 6D–6G) assay, cell viability, migration, and invasion were reduced by silencing SOX2, but the addition of miR-222-5p mimic abolished these effects of SOX silencing on the above cell functions. These results suggest that SOX2 downregulation attenuated cell proliferation, migration, and invasion by regulating miR-222-5p expression in HepG2 cells.

### Silencing of SOX2 inhibits HCC tumor growth in nude mice by activating CCAT1, EGFR, miR-222-5p and downregulating CYLD

**Figure 7:**
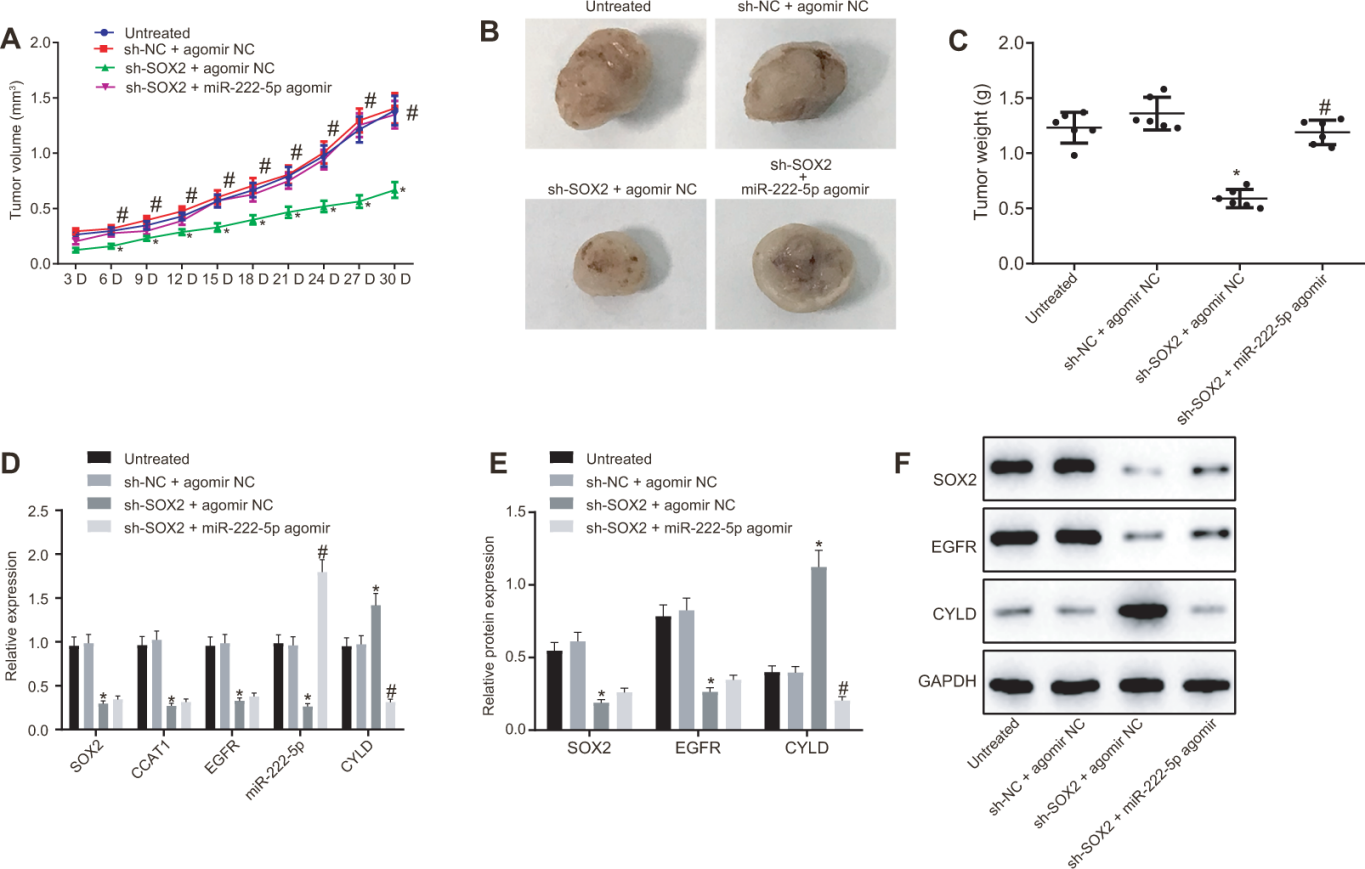
**SOX2 silencing represses tumor growth in mice with HCC by activating CCAT1, EGFR, miR-222-5p and by downregulating CYLD.** HepG2 cells expressing sh-SOX2, miR-222-5p agomir or corresponding controls (sh-NC or agomir-NC) were administrated subcutaneously to nude mice for developing *in vivo* tumor model. (**A**) Tumor growth rate in nude mice. (**B**) Representative images showing tumor mass collected from nude mice at day 30. (**C**) Tumor weights in nude mice. (**D**) mRNA expression of SOX2, EGFR and CYLD and CCAT1 and miR-222-5p expression in nude mice xenografts determined by RT-qPCR normalized to U6 and β-actin. (**E**, **F**) Protein expression of SOX2, EGFR and CYLD in nude mice xenografts determined by western blot analysis normalized to GAPDH. n = 6/group. * *p* < 0.05 *vs.* untreated mice; # *p* < 0.05 *vs.* mice treated with HepG2 cells expressing sh-SOX2 + agomir NC. Data were expressed as mean ± standard deviation. Data from multiple groups were compared by one-way ANOVA and Tukey’s *post hoc* test. Data were compared between groups at different time points by repeated measures ANOVA and Bonferroni *post hoc* testing.

**Figure 8:**
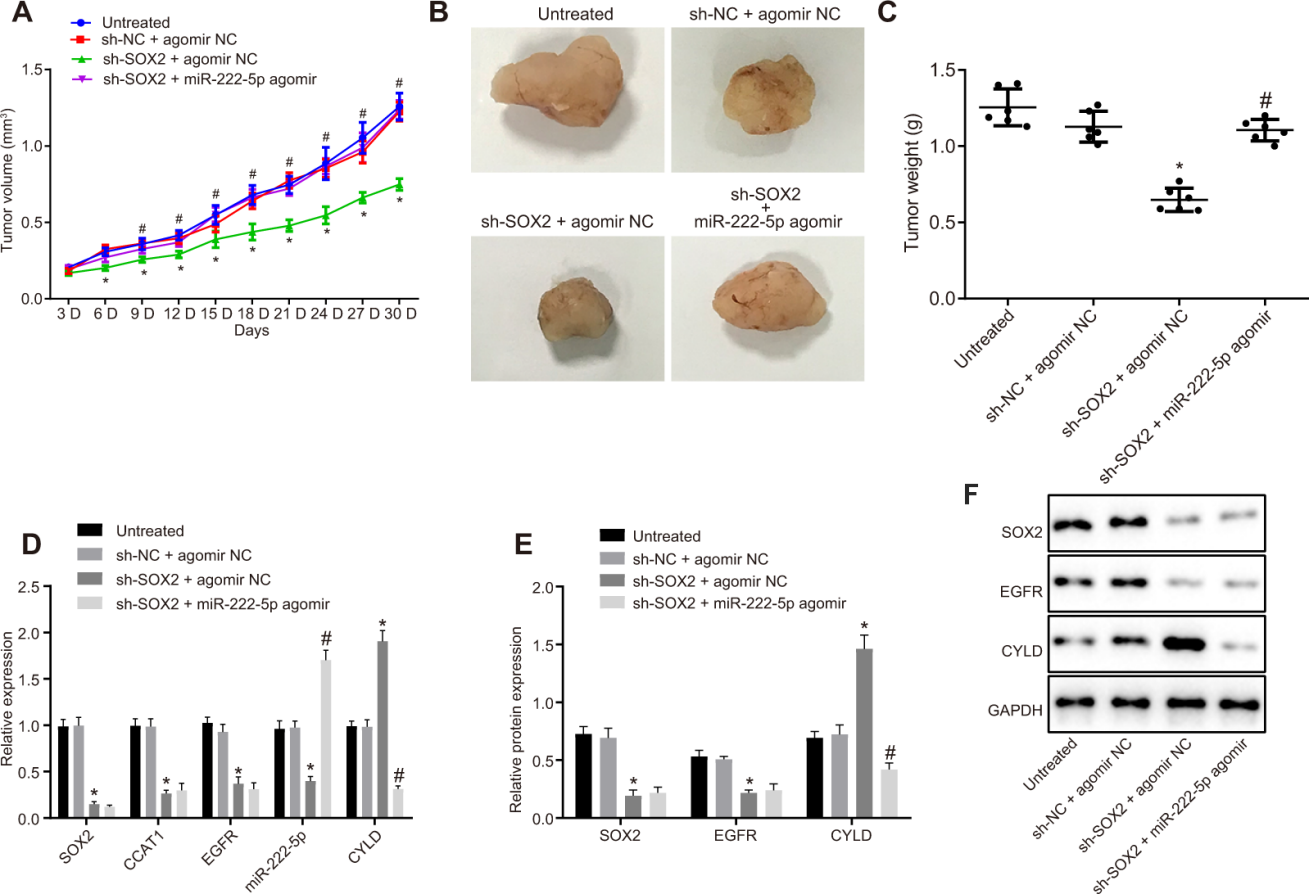
**Loss of SOX2 inhibits tumor growth in mice with HCC by activating CCAT1, EGFR, miR-222-5p and downregulating CYLD.** Huh7 cells expressing sh-SOX2, miR-222-5p agomir or corresponding controls (sh-NC or agomir-NC) were administrated subcutaneously to nude mice for developing the *in vivo* tumor model. (**A**) Tumor growth rate in nude mice presented as tumor volume. (**B**) Representative images showing tumor mass collected from nude mice at day 30. (**C**) Terminal tumor weights in nude mice. (**D**) mRNA expression of SOX2, EGFR and CYLD and CCAT1 and miR-222-5p expression in nude mice xenografts determined by RT-qPCR normalized to U6 and β-actin. (**E**, **F**) Protein expression of SOX2, EGFR and CYLD in nude mice xenografts determined by western blot analysis normalized to GAPDH. n = 6/group. * *p* < 0.05 *vs.* untreated mice; # *p* < 0.05 *vs.* mice treated with Huh7 cells expressing sh-SOX2 + agomir NC. Data were expressed as mean ± standard deviation. Data from multiple groups were compared by one-way ANOVA and Tukey’s *post hoc* test. Data were compared between groups at different time points by repeated measures ANOVA and Bonferroni’s *post hoc* testing.

Subsequently, we tested the effect of SOX2 on tumor growth in nude mice bearing HepG2 cell tumors expressing sh-SOX2, miR-222-5p agomir or corresponding controls (sh-NC or agomir-NC). The nude mice bearing HepG2 cells expressing sh-SOX2 showed significantly reduced tumor growth, volume, and weight, which were restored by the addition of miR-222-5p agomir (Figure 7A–7C). Additionally, sh-SOX2 treatment decreased mRNA expression of SOX2, CCAT1, EGFR, and miR-222-5p in tumor isolated from nude mice, but increased the mRNA expression of CYLD. However, additional treatment of miR-222-5p agomir increased miR-222-5p but reduced CYLD expression, while having no effects on the other genes under consideration (Figure 7D). Furthermore, protein expression of SOX2 and EGFR was reduced while there was a greater increase in CYLD expression following treatment with sh-SOX2, while additional miR-222-5p agomir only decreased CYLD protein expression without affecting SOX2 and EGFR expression (Figure 7E, 7F). To confirm further the above-indicated findings, we also conducted analogous experiments in Huh7 cells, which showed the same results as seen in HepG2 cells (Figure 8). In conclusion, SOX2, CCAT1 and EGFR proved to be upstream of miR-222-5p such that SOX2 downregulation repressed HCC cell progression and increased CYLD expression through downregulating CCAT1, EGFR, and miR-222-5p.

## DISCUSSION

HCC affects millions of people worldwide each year, bringing a devastating mortality rate. Yet, the cellular mechanisms underlying HCC are scantly understood. In the study, we explored a mechanism leading to progression of HCC *in vitro* and in tumor bearing mice. Among the key findings, SOX2 expression proved to be increased in human HCC tissues, which was confirmed in six different human HCC cell lines. Second, CCAT1 was downstream of SOX2, and was upregulated by SOX2 in HCC. Third, SOX2 silencing had a suppressive effect on HCC cell proliferation, migration, and invasion, and therefore protected against the progression of HCC. Fourth, and most mechanistically most importantly, the effects of SOX2 seemingly work through upregulation of CCAT1, which in turn leads to upregulation of EGFR. Increased expression of EGFR contributed to upregulation of miR-222-5p, which targeted and inhibited CYLD expression. The discovery of this signaling pathway with critical involvement in the progression of HCC serves as the first step for better understanding of HCC, and hopefully improved treatment.

We found increased SOX2 expression in HCC tissues in our study, which is in line with previous studies [14, 15]. Moreover, we found that SOX2 was involved in HepG2 cell proliferation, migration, and invasion, also in agreement with a previous study [9]. Furthermore, for the first time, we found that CCAT1 was regulated the progression of HCC by acting as a downstream signaling molecule of SOX2. Although SOX2 and CCAT1 have already been implicated in squamous cell carcinoma, this is the first such finding in HCC [22]. CCAT1 has been previously documented to promote HCC growth [20, 21], and to bind at the super-enhancer of EGFR to upregulate EGFR expression by forming a complex with TP65 and SOX2 [22]. EGFR is a known enhancer of HCC proliferation and invasiveness [26, 27]. Although the present results might be viewed as largely confirmatory, we for the first time show that the members of the pathway are functionally linked to promote the progression of HCC.

We found that EGFR promoted the expression of miR-222-5p in HCC. This result is, to the best of our knowledge, a novel finding, supported indirectly by two previous observations; first, EGFR-mediated functions in cancer cells involve other microRNAs [28, 29], and second, miR-222-5p expression is increased in HCC [32]. Therefore, we feel that the binding relationship between EGFR and miR-222-5p in HCC and other cancers merits further, more detailed investigation. Another novel finding in this study was our identification of CYLD as the direct target gene of miR-222-5p. We note that decreased CYLD expression was found previously in HCC tissues [36], and that another miR, namely miR-922, has been shown to target CYLD in HCC [37]. miR-922 targets and inhibits CYLD expression, leading to promotion of c-Myc and cyclin D1 as well as cancer cell proliferation, thus presenting another possible mechanism for HCC promotion. More importantly, a previous study found that miR-501 promoted HCC progression in Han Chinese by enhancing cell proliferation via regulation of CYLD [38]. We speculate that CYLD could function to deubiquitinate specific substrates in signaling pathways such as NF-κB and JNK, which are well-known to be involved in promoting cancers [39–41]. Moreover, mice lacking functional CYLD have increased rates of cancer progression [42]. These studies, together with present results, implicate CYLD as a cancer suppressor which prove to be protective against HCC [39], thus presenting a new therapeutic target.

There are a few notable limitations of the current study. First, although HCC accounts for 80% of primary liver cancer, these results likely do not generalize to other forms of liver cancer. Second, we chose to use HepG2 cells in most of our studies because of its high constitutive SOX2 expression. Although the other human HCC cell lines also had elevated expression of SOX2, HepG2 may not be the only representative of all forms of HCC with respect to the importance of this pathway. Therefore, results from this study should be confirmed in other human cancer cell lines. Although we unmasked the involvement of CCAT1, EGFR, miR-222-5p and CYPD in HCC progression, we cannot prove entirely that they constitute a single signaling pathway. Further experiments could identify and confirm the complete upstream-downstream relationship of these signaling molecules. Although we did establish binding relationships between SOX2 and CCAT1 and between miR-222-5p and CYLD, we did not prove a corresponding binding relationship between EGFR and miR-222-5p. Due to the importance of this signaling pathway in HCC, we plan to make a more detailed target gene binding experiment.

In conclusion, SOX2-mediated progression of HCC involves the upregulation of CCAT1, EGFR, and miR-222-5p, which together targets and inhibits CYLD. This novel signaling pathway merits further investigation that may lead to better understanding of and treatment for HCC.

## MATERIALS AND METHODS

### Study subjects

HCC tissues and their matched para-cancerous tissues were collected from 68 patients (25-71 years old; 48 males and 20 females; > 50 years old, 23 cases; ≤50 years old, 45 cases) who were pathology-confirmed as having primary HCC in Affiliated Hospital of Youjiang Medical University for Nationalities from April 2015 to December 2017. There were 26 cases at stage I-II and 42 at stage III according to the Edmondson pathological grade (World Health Organization). Tumor-node-metastasis (TNM) staging (7^th^ edition; American Joint Committee on Cancer) revealed 17 cases at stage I-II and 51 cases at stage IIIa, and 21 cases of lymph node metastasis and 47 cases of non-lymph node metastasis. The enrolled HCC patients had not received radio-, chemo- or immune- therapy before surgery. Normal liver tissues were obtained from 28 healthy individuals (25-71 years old; 16 males and 12 females) who underwent abdominal surgery for reasons unrelated to hepatocarcinoma lesions at the same hospital. The collected tissues were stored at -80° C until use.

### Culture of human HCC cell lines

Human HCC cell lines (HepG2, MHCC-97H, MHCC-97L, SMMC7721, Hep3B, and Huh7) and a normal human liver cell line LO2 (Chinese Academy of Sciences cell bank, Shanghai, China) were maintained in RPMI 1640 medium supplemented with penicillin (50 U/mL), streptomycin (50 μg/mL), L-glutamine (300 μg/mL), and 10% fetal bovine serum (FBS; Sigma-Aldrich, Taufkirchen, Germany). Twenty percent of the medium was replaced every three days.

### HepG2 cell transfection

HepG2 cells were cultured in RPMI 1640 medium containing 10% FBS at 37° C with 50 mL/L CO_2_. When cells reached 90% confluence and were in the logarithmic growth phase, cells were trypsinized to prepare cell suspension (2.5 × 10^4^ cells/mL), which was seeded into a 6-well plate at 2 mL/well. When cells reached 30% confluence and were in the logarithmic growth phase, different lentivirus was added (2 × 10^6^ TU lentiviruses were mixed with 5 μg of polybrene and 1 mL serum-free and antibacterial-free medium). Transfection was observed under an inverted fluorescence microscope after two or three days. After transfection for two days, 1 μg/mL of puromycin was added to each well to screen stably transfected cells. Cells were then allowed to grow in culture to obtain stable transfected cells. After successful lentivirus infection, cells at 70-80% of confluence were selected and subjected to transient transfection of miR-222-5p mimic or inhibitor using the liposome Lipofectamine^TM^ 2000 kit as per instructions (Invitrogen, Carlsbad, CA, USA). After two days, cells were harvested for subsequent experiments.

Plasmids, and lentivirus clone vectors pLKO.1-TRC (plasmid #10878) and pcDNA3.3-SOX2 (plasmid, #26817) were provided by Addgene (Cambridge, MA, USA). Double-stranded oligonucleotide short hairpin RNA (shRNA) was cloned at the AgeI/EcoRI site in the pLKO.1-TRC lentiviral vector [22]. HepG2 cells were treated with overexpression empty lentivirus vector [overexpression-negative control (oe-NC)], sh-NC lentivirus, sh-SOX2 lentivirus, sh-CCAT1 lentivirus, oe-CCAT1 lentivirus, oe-SOX2 lentivirus, oe-EGFR-1 lentivirus at doses of 0.5, 1.0, and 1.5 μg, 1.5 μg oe-EGFR-3 lentivirus, DMSO, 1 μM gefitinib (EGFR-inhibitor), mimic-NC, miR-222-5p mimic, inhibitor-NC, miR-222-5p inhibitor, sh-EGFR lentivirus, small interfering (si)-NC lentivirus, and si-CYLD lentivirus, alone or in combination. After 48 hours of transient transfection, expression levels in HepG2 cells were detected by reverse transcription quantitative polymerase chain reaction (RT-qPCR) to evaluate transfection efficiency.

### Transplantation of HCC cells into nude mice

Male nude mice (n = 24, 4-6 weeks old, 17-20 g) of specific pathogen-free (SPF) grade were injected with untreated HepG2, or HepG2 cells stably transfected with sh-NC + agomir-NC, sh-SOX2 + agomir NC or sh-SOX2 + miR-222-5p agomir (lentivirus vector and reagents used were purchased from GenePharma, Shanghai, China) (n = 6). Before cell transplantation, cells were mixed with Matrigel at a 1:1 v/v ratio, and 0.2 mL portions of suspension (5 × 10^6^ cells/mL) were inoculated subcutaneously to the right armpit of the nude mice. Body weight and tumor size of the mice were measured every three days. Tumor size was calculated based on the formula: V (mm^3^) = 1/2L × D^2^, where L is the longest axis and D the shortest, and the growth curve was plotted. Nude mice were euthanatized after three weeks by the CO_2_ exposure, and the tumors were removed, weighed, and imaged.

### Dual luciferase reporter assay

TargetScan (http://www.targetscan.org/vert_71/) was used to predict the targeted binding site between miR-222-5p and CYLD, which was then verified by dual luciferase reporter assay. HEK-293T cells (cell bank of Shanghai Institute of Chinese Academy of Sciences, Shanghai, China) were cultured in Dulbecco’s Modified Eagle Medium (DMEM). Upon reaching 80-90% confluence, cells were detached with 0.25% trypsin and cultured in a 5% CO_2_ incubator at 37° C. Experiments were performed in cells at the logarithmic growth phase. A synthetic CYLD 3'-untranslated region (UTR) gene fragment was introduced to the pGL3-control vector (Promega, Madison, WI, USA) at the XhoI and BamH I endonuclease sites. The mutation site of the seed sequence (MUT) was imposed on wild type CYLD (WT) gene by restriction endonucleases. Then the MUT fragment was inserted into the pGL3-control vector using T4 DNA ligase. Luciferase reporter plasmids WT and MUT were co-transfected with miR-222-5p mimic into HEK-293T cells. After 48 hours of transfection, cells were harvested and lysed. Luciferase intensity was measured by a Luminometer TD-20/20 detector (E5311, Promega) using the Dual-Luciferase® R Reporter Assay System Kit (Promega).

### 3-(4, 5-dimethylthiazol-2-yl)-2, 5-diphenyltetrazolium bromide (MTT) assay

Cells were collected for trypsinization after transfection for 24 hours, and seeded into a 96-well plate (4 × 10^3^ cells/well) containing 200 μL of complete medium and 20 μL of MTT (5 mg/mL) in each well. Cells were incubated at 37° C with 5% CO_2_ for four hours. Absorbance was measured at 450 nm by a MTT enzyme-linked immunometric meter (US 6111636, Thermo Fisher, Waltham, MA, USA).

### Cell invasion and migration determined by Transwell assay

Cell migration was measured in a Matrigel-free chamber (Merck Millipore, Darmstadt, Germany) while cell invasion was measured by a Matrigel-containing chamber (BD Biosciences, Franklin Lakes, NJ, USA), closely following methods in our previously published study [43]. Cells were imaged with a microscope, and ten to twenty fields were randomly selected for cell counting.

### Protein expression determined by western blot analysis

Human tissues or transfected cells were lysed with radio-immunoprecipitation assay lysis and extraction buffer (Thermo Fisher Scientific). Protein concentration was estimated by a Bio-Rad Protein Assay Kit (Bio-Rad, Hercules, CA, USA). Proteins were separated by sodium dodecyl sulfate polyacrylamide gel electrophoresis and then transferred to a nitrocellulose membrane (Bio-Rad). Membranes were incubated with primary rabbit antibodies (Abcam, Cambridge, UK) against SOX2 (ab97959), EGFR (ab40815), CYLD (ab137524), and β-actin (ab8226) overnight at 4° C, and then re-probed with secondary antibody at room temperature for one hour. An ECL fluorescence detection kit (Cat. No. BB-3501, Amersham, UK) was used to visualize protein bands, which were imaged by the Bio-Rad Image Analysis System (Bio-Rad) and analyzed by Quantity One v4.6.2 software, using β-actin as internal reference.

### mRNA expression determined RT-qPCR

Total RNA was extracted by RNeasy Mini Kit (Qiagen, Valencia, CA, USA). cDNA was produced in mRNA by reverse transcription kits (RR047A, Takara, Tokyo, Japan). cDNA was also obtained from miRNA using a miRNA First Strand cDNA Synthesis tailing reaction kit (B532451-0020, Sangon, Shanghai, China). Samples were loaded using a SYBR® Premix Ex TaqTM II (Perfect Real Time) kit (DRR081, Takara) and the RT-qPCR reaction was performed by an ABI 7500 system (ABI, Foster City, CA, USA) with GAPDH and U6 serving as internal references. The universal negative primer for miR and the upstream primer for U6 were provided in the miRNA First Strand cDNA Synthesis tailing reaction kit, and other primers were synthesized by Sangon (Table 1). Target gene expression was calculated by the 2^-ΔΔCt^ method.

**Table 1:** Primer sequences for RT-qPCR.

**Genes**	**Forward (3’-5’)**	**Reverse (3’-5’)**
SOX2	GGATAAGTACACGCTGCCCG	ATGTGCGCGTAACTGTCCAT
CCAT1	CATTGGGAAAGGTGCCGAGA	ACGCTTAGCCATACAGAGCC
EGFR	GCCGGAGTCCCGAGCTA	CCGGCTCTCCCGATCAATAC
CYLC	TGCCTTCCAACTCTCGTCTTG	AATCCGCTCTTCCCAGTAGG
β-actin	GCGAGAAGATGACCCAGGATC	CCAGTGGTACGGCCAGAGG
U6	CTCGCTTCGGCAGCACA	AACGCTTCACGAATTTGCGT
miR-222-5p	GGGCTCAGTAGCCAGTGTA	CAGTGCGTGTCGTGGAGT

### Electrophoretic mobility shift assay (EMSA)

EMSA was performed by a LightShift Chemiluminescent EMSA kit (Thermo Scientific, Waltham, MA, USA). In briefly, the non-specific interactions were minimized using a binding reaction mixture (20 μL) supplemented with 25 mM N-2-hydroxyethylpiperazine-N'-2-ethanesulfonic acid (HEPES)-KOH (pH = 7.5), 100 mM potassium chloride (KCI), 0.1 mM ethylene diaminetetra acetic acid (EDTA), 17% glycerol, 1 mM dithiothreitol (DTT), 50 ng of purified protein, ~1 pmol of labeled probe, competitor DNA (25, 100 or 400 pmoL), and 4 mg of poly (dI-dC). The mixtures were incubated for 25 min at 22 ° C. Biotin-labeled DNA was detected using the chemiluminescence method according to the instructions of a ChemiDoc™ MP Imaging System (Bio-Rad, Hercules, CA, USA).

### Chromatin immunoprecipitation (ChIP)-qPCR analysis

Cells were crosslinked with 1% formaldehyde solution and neutralized by 1.25 M glycine. Crosslinked cells were then lysed and sonicated with a Bioruptor (Diagenode). Sonicated chromatin was precleared with Dynabeads protein A/G and then incubated with indicated antibody for overnight at 4° C. Afterwards, the sample was incubated with beads for a further two hours. DNA was eluted from immunoprecipitate complexes, reverse crosslinked, and purified with QIAquick PCR spin kit (QIAGEN). ChIP-qPCR was performed to verify the ChIP-seq results, with the gene desert (chr11:127,277,673–127,322,674) serving as a negative control.

### Immunohistochemical staining

Embedded tissue samples were dewaxed by xylene, hydrated by gradient alcohol (anhydrous ethanol, 95% ethanol, 75% ethanol for 3 min), and subjected to antigen retrieval. After that, the samples were added with 50 μL of 3% H_2_O_2_ for incubation for 20 minutes at room temperature to remove endogenous peroxidase activity. Tissue samples were then incubated with primary rabbit antibodies (Abcam) against EGFR (ab52894) and CYLD (ab137524) at 4° C overnight. Rabbit serum was used instead of primary antibody as a NC. Tissues were cultured with 50 μL polymer enhancer at 37° C for 20 minutes and with enzyme-labeled rabbit anti-polymer (50 μL) at 37° C for 30 min. Following that, two drops or approximately 100 μL of freshly prepared diaminobenzidine was added to each tissue section followed by observation under a microscope for 3-10 min. Presence of brownish-yellow stain was regarded as positive. Subsequently, tissue samples were counterstained with hematoxylin, dehydrated with gradient alcohol (75% ethanol, 95% ethanol and absolute ethanol), sealed with neutral resin, and observed under a microscope. Positive staining for EGFR or CYLD was brownish-yellow fine particles in cells. We used a scoring system where ≤ 10% positive staining was designated as negative (0 points), regardless of the intensity of staining, 11-51% staining was 2 points, 51-81% was 3 points, and ≥ 81% was 4 points. Staining intensity was graded as follows: 1 point for weak intensity; two points for moderate intensity, and three points for high intensity. The two scores were added as a total score, where 0 points was considered negative (-), 3 points as weakly positive (+), 4-5 points moderately positive (++), and 6-7 points as strongly positive (+++).

### Statistical analysis

SPSS version 21.0 (IBM Corp. Armonk, NY, USA) was used for statistical analysis. Data were expressed by mean ± standard deviation. If conforming to normal distribution and homogeneity of variance, unpaired data between two groups were compared by unpaired *t*-test. Data comparisons among multiple groups were performed using one-way analysis of variance (ANOVA) and Tukey *post hoc* test. Data comparisons between groups at different time points were performed by repeated measures ANOVA, followed by Bonferroni post hoc test. Differences were considered significant when *p* < 0.05.

### Ethics approval and consent to participate

The clinical experiments in this study were approved by ethics committee of Affiliated Hospital of Youjiang Medical University for Nationalities. Ethics agreements was obtained from the tissue donors or their relatives by written informed consent. The experiments involved animals were implemented according to the principles embodied in the National Institutes of Health Guide for the Care and Use of Laboratory. Efforts were made to avoid all unnecessary distress to the animals.
